# Supplementary material for: Seismological expression of the iron spin crossover in ferropericlase in the Earth’s lower mantle
Source: Nat Commun. 2021 Oct 8;12:5905. doi: 10.1038/s41467-021-26115-z (PMC8501025; doi:10.1038/s41467-021-26115-z)
Supplement: Supplementary file 1 — Supplementary Information [file 41467_2021_26115_MOESM1_ESM.pdf]

Supplementary Information: Seismological Expression of the Iron Spin Crossover in Ferropericlase in the Earth's Lower Mantle

Grace E. Shephard*[1], Christine Houser [2], JohnW. Hernlund [2], Juan J. Valencia-Cardona [3], Reidar G. Trønnes [1,4], Renata M. Wentzcovitch [2,5,6]

1. Centre for Earth Evolution and Dynamics (CEED), Department of Geosciences, University of Oslo, Norway
2. Earth-Life Science Institute, Tokyo Institute of Technology, Tokyo, Japan
3. Department of Chemical Engineering and Material Science, University of Minnesota, Twin Cities, USA
4. Natural History Museum, University of Oslo, Norway
5. Department of Earth and Environmental Sciences, Columbia University, Lamont-Doherty Earth Observatory, Palisades, USA
6. Department of Applied Physics and Applied Mathematics, Columbia University, New York City, USA

Supplementary Figures

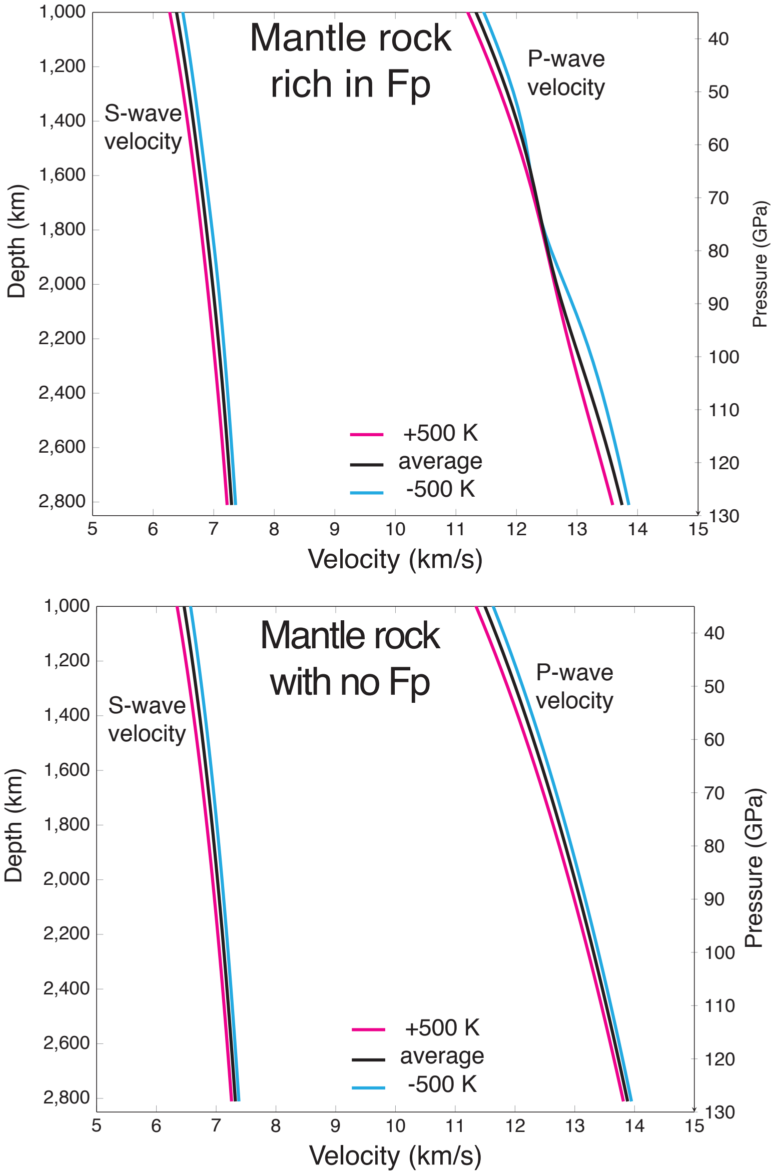

Supplementary Figure 1

Calculated shear and compressional velocities using the ab initio technique described in Methods section for harzburgite (top) and an Fp-free model composition (bottom). The ab initio calculations were performed for each composition with the self consistent geotherm anchored at 1873 K at the top of the lower mantle (black) along with the same calculation anchored at 500 K below (blue) and a 500 K above (magenta). The properties of individual minerals were calculated using an ideal solid solution formalism, where the end-members were the Mg-compound and a (Mg,Fe) solid solution with Mg = 0.875 and Fe = 0.125 for bridgmanite [1] and Mg = 0.8125 and Fe = 0.1875 for ferropericlase [2]. Aggregate elastic properties were obtained using the Voigt-Reuss-Hill average for the Fp-free mantle model composition, consisting of 94 wt.% bridgmanite and 6 wt.% calcium perovskite and Fp-rich harzburgite consisting of 74 wt.% bridgmanite, 24 wt.% ferropericlase, and 2 wt.% calcium perovskite

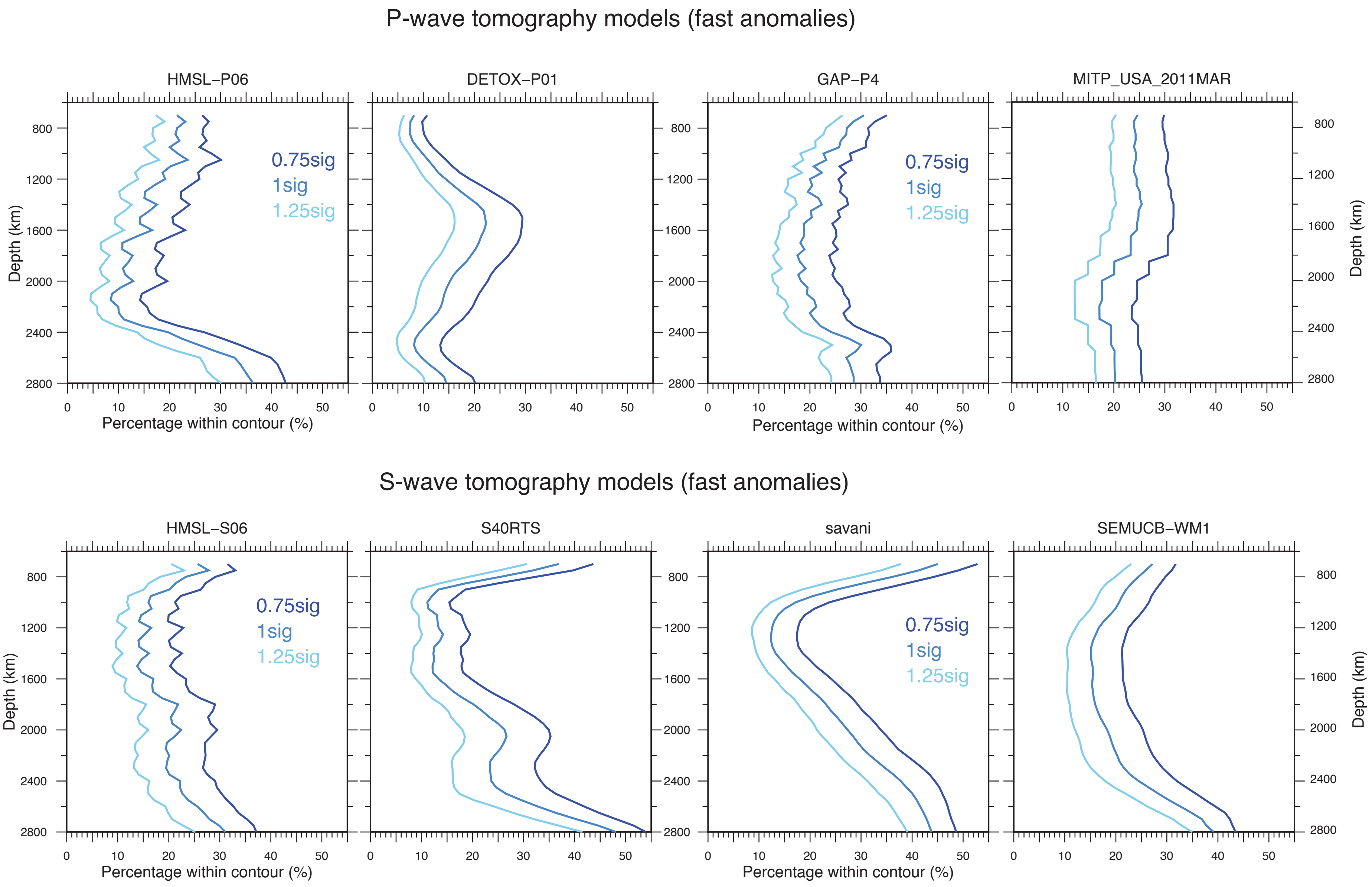

P-wave tomography models (fast anomalies)
HMSL−P06
DETOX−P01
GAP−P4
MITP_USA_2011MAR
0.75sig
1sig
1.25sig
Depth (km)
800
1200
1600
2000
2400
2800
0
10
20
30
40
50
Percentage within contour (%)
S-wave tomography models (fast anomalies)
HMSL−S06
S40RTS
savani
SEMUCB−WM1

Supplementary Figure 2 (previous page)
Surface area coverage for individual models and for variable sigma contours, for fast anomalies. Area fraction of fast (>+0.75, >+1, >+1.25 σ) anomalies as a function of depth for the 8 tomography models individually. This is similar to Fig. 1b and 4a (and Supplementary Fig. 5 for $HMSL_3$), but presented here for the individual tomography models (4 P-wave and 4 S-wave) prior to being summed into the vote map. There is high variability between and within the individual tomography models, including the joint HMSL models. While a decorrelation between P- and S-wave models is apparent between some model combinations, it is not in others, which is why a single pair of tomography models in isolation may not render a robust signal of the spin transition. Other features in these models, such as the oscillatory behaviour in HMSL, are not well understood and can also distract from the broader trends in P-wave and S-wave velocity.

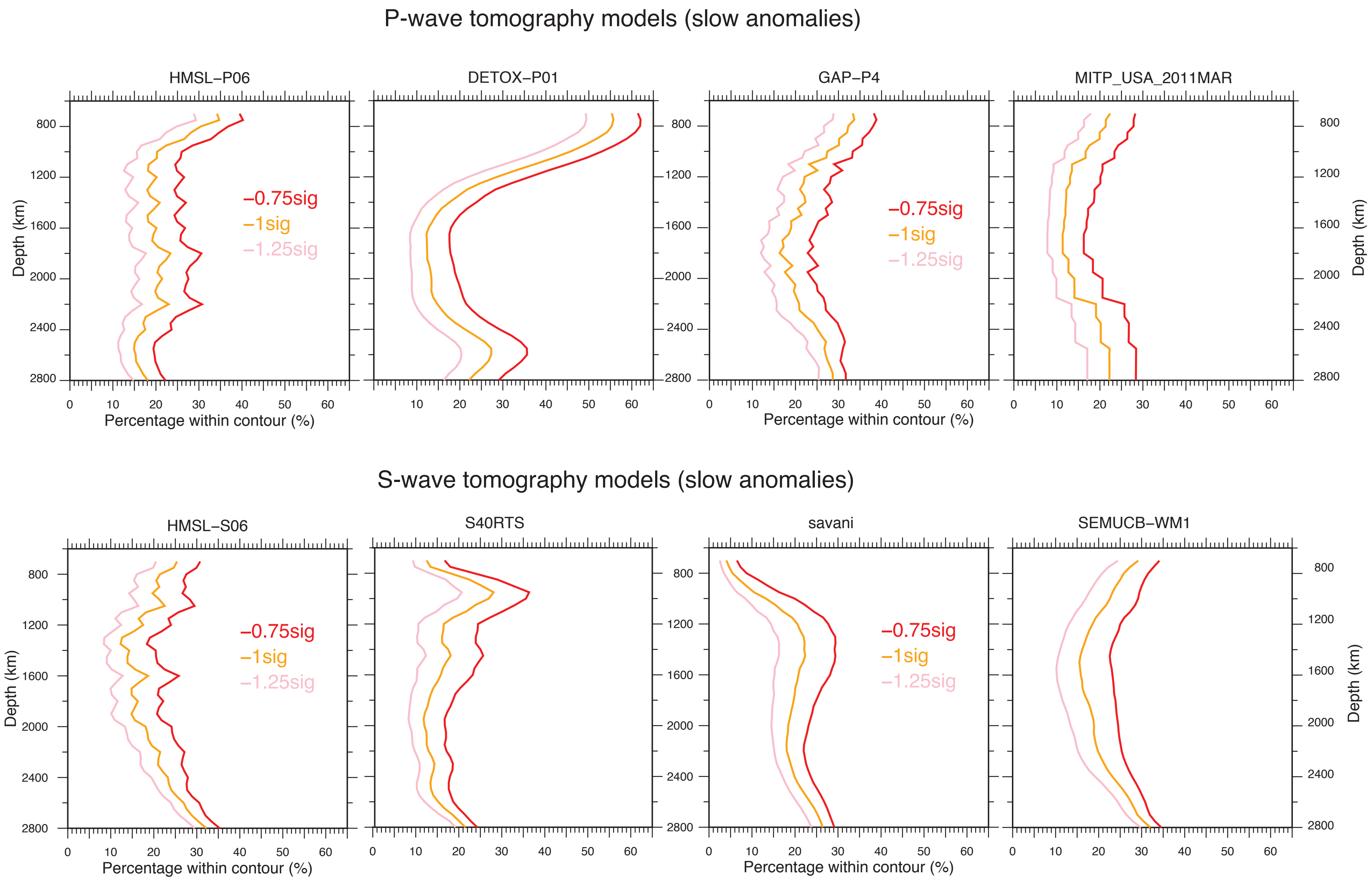

P-wave tomography models (slow anomalies)
HMSL–P06
DETOX–P01
GAP–P4
MITP_USA_2011MAR
–0.75sig
–1sig
–1.25sig
Depth (km)
Percentage within contour (%)
S-wave tomography models (slow anomalies)
HMSL–S06
S40RTS
savani
SEMUCB–WM1

Supplementary Figure 3 (previous page)
Surface area coverage for individual models and for variable sigma contours, for slow anomalies. Area fractions of slow (<-0.75, <-1, <-1.25 σ) anomalies as a function of depth for the 8 tomography models. Similar to Fig. 1c, but here presented for the individual tomography models (4 P-wave and 4 S-wave) prior to being summed into the vote map. As with the fast anomalies (Supplementary Figure 2), there is high variability between the tomography models. Other features in these models, such as the oscillatory behaviour in HMSL, are not well understood and can also distract from the broader trends in P-wave and S-wave velocity.

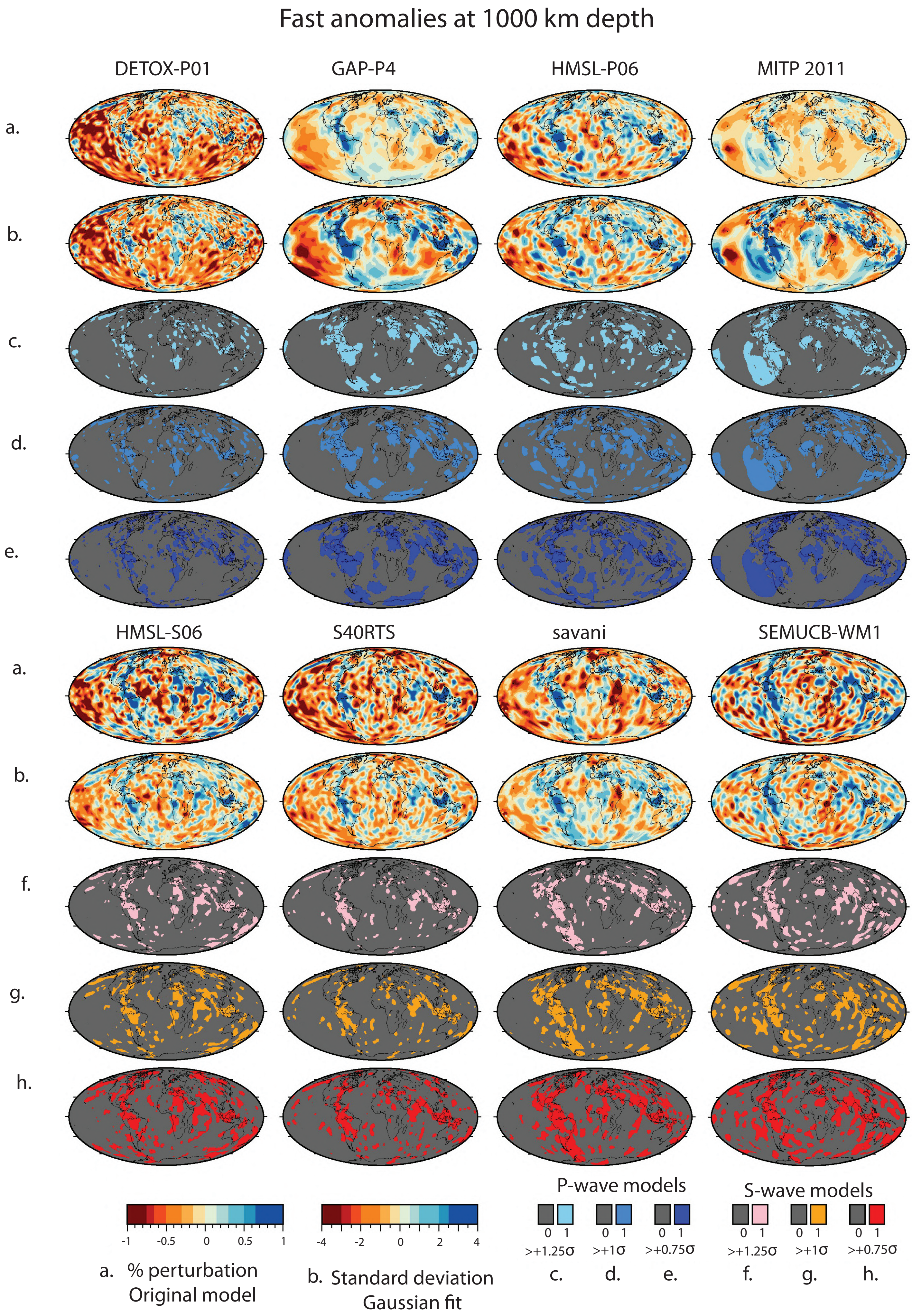

Fast anomalies at 1000 km depth
DETOX-P01
GAP-P4
HMSL-P06
MITP 2011
a.
b.
c.
d.
e.
HMSL-S06
S40RTS
savani
SEMUCB-WM1
a.
b.
f.
g.
h.
-1
-0.5
0
0.5
1
a. % perturbation
Original model
-4
-2
0
2
4
b. Standard deviation
Gaussian fit
P-wave models
0 1
>+1.25σ
>+1σ
>+0.75σ
c.
d.
e.
S-wave models
f.
g.
h.

Supplementary Figure 4 (previous page)
A graphical example figure of the contouring procedure for fast anomalies at 1000 km depth. This step occurs before the models are added into vote maps and are equivalent to what is shown in Supplementary Figures 2-3, and Figure 3 Steps 1-3. Each of the 8 tomography models are shown in their original format (panel a) and after the gaussian fitting is applied to 1000-2200 km depth (panel b; See Supplementary Figure 10). The models are contoured for values equal to or higher than +0.75, +1, +1.25 σ) anomalies for the P-wave models (panels c, d, e) and S-wave models (panels f, g, h), respectively. The fast vote maps in the main manuscript were constructed from the >+1 sigma vote maps. Shephard et al. (2017)[4] presents further details of the vote map methodology.

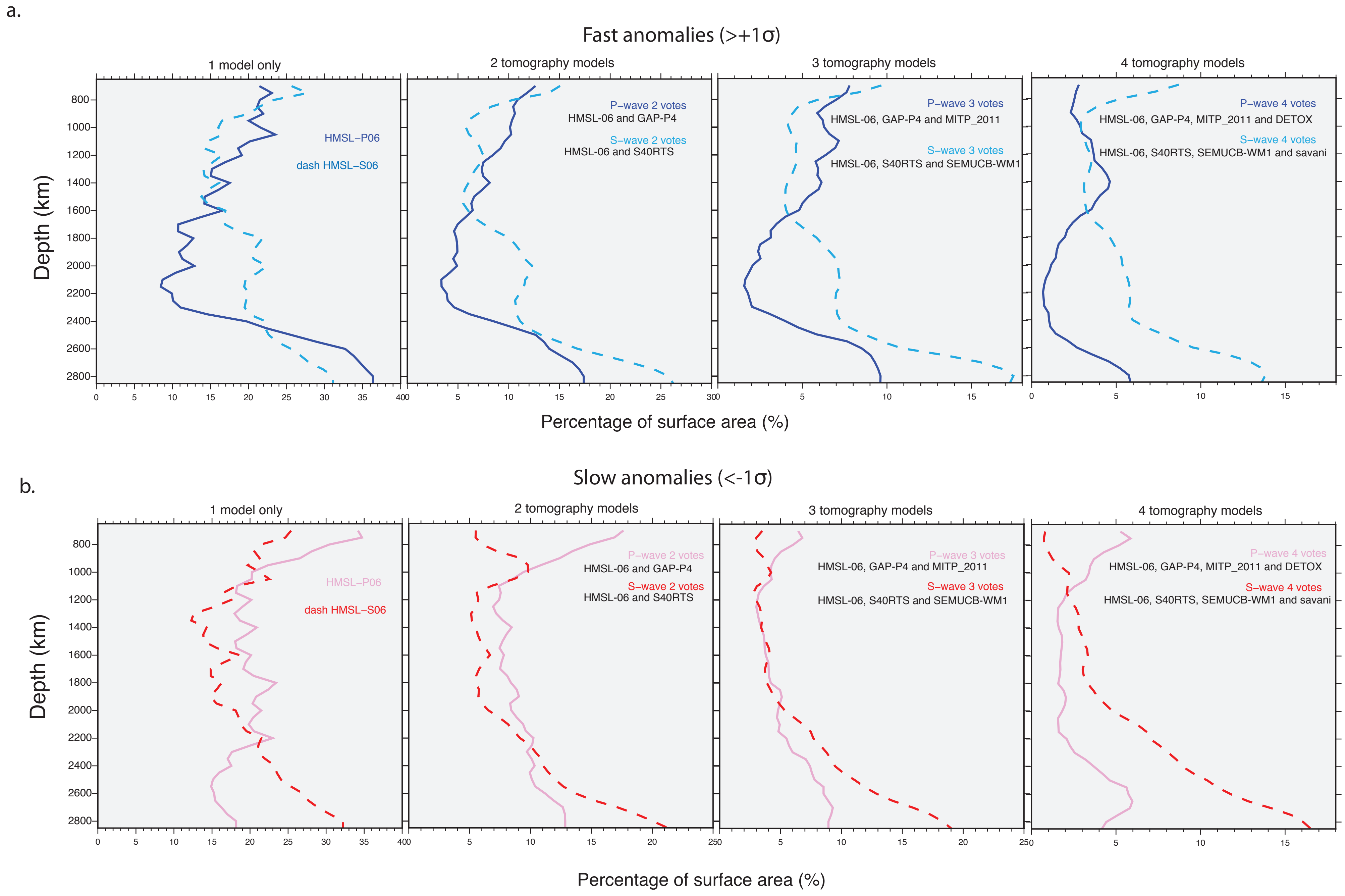

a.
Fast anomalies (>+1σ)
1 model only
2 tomography models
3 tomography models
4 tomography models
HMSL–P06
dash HMSL–S06
P–wave 2 votes
HMSL-06 and GAP-P4
S–wave 2 votes
HMSL-06 and S40RTS
P–wave 3 votes
HMSL-06, GAP-P4 and MITP_2011
S–wave 3 votes
HMSL-06, S40RTS and SEMUCB-WM1
P–wave 4 votes
HMSL-06, GAP-P4, MITP_2011 and DETOX
S–wave 4 votes
HMSL-06, S40RTS, SEMUCB-WM1 and savani
Depth (km)
Percentage of surface area (%)
b.
Slow anomalies (<-1σ)
1 model only
2 tomography models
3 tomography models
4 tomography models
HMSL–P06
dash HMSL–S06
P–wave 2 votes
HMSL-06 and GAP-P4
S–wave 2 votes
HMSL-06 and S40RTS
P–wave 3 votes
HMSL-06, GAP-P4 and MITP_2011
S–wave 3 votes
HMSL-06, S40RTS and SEMUCB-WM1
P–wave 4 votes
HMSL-06, GAP-P4, MITP_2011 and DETOX
S–wave 4 votes
HMSL-06, S40RTS, SEMUCB-WM1 and savani
Depth (km)
Percentage of surface area (%)

Supplementary Figure 5 (previous page)
The influence of using fewer tomography models. Area coverage as a function of depth for the sequential addition of tomography models used in this study. P-wave models (solid) and S-wave models (dashed), top panels (a) show fast anomalies, and bottom (b) panels show slow anomalies. Models used are listed within each panel (the HMSL profiles (1 model) are the same as shown in Supplementary Figures 2 and 3). There is an apparent decorrelation of P-wave and S-wave profiles in all combinations. However, the signal becomes more apparent when the models are summed into the vote maps, which identify the most common features between tomography models.

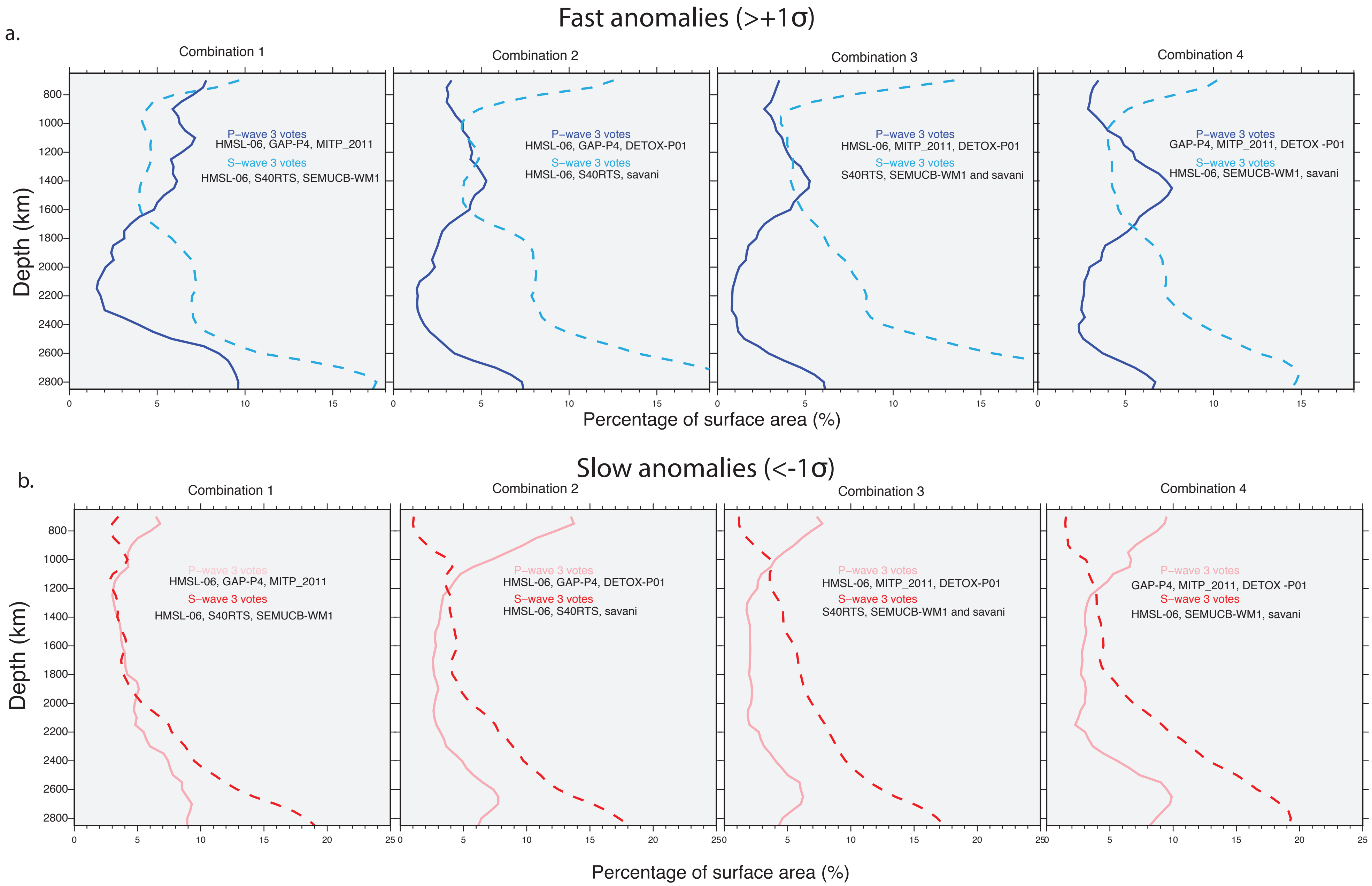

Fast anomalies (>+1σ)
a.
Combination 1
Combination 2
Combination 3
Combination 4
Depth (km)
P–wave 3 votes
HMSL-06, GAP-P4, MITP_2011
S–wave 3 votes
HMSL-06, S40RTS, SEMUCB-WM1
P–wave 3 votes
HMSL-06, GAP-P4, DETOX-P01
S–wave 3 votes
HMSL-06, S40RTS, savani
P–wave 3 votes
HMSL-06, MITP_2011, DETOX-P01
S–wave 3 votes
S40RTS, SEMUCB-WM1 and savani
P–wave 3 votes
GAP-P4, MITP_2011, DETOX -P01
S–wave 3 votes
HMSL-06, SEMUCB-WM1, savani
Percentage of surface area (%)
Slow anomalies (<-1σ)
b.
Combination 1
Combination 2
Combination 3
Combination 4
Depth (km)
P–wave 3 votes
HMSL-06, GAP-P4, MITP_2011
S–wave 3 votes
HMSL-06, S40RTS, SEMUCB-WM1
P–wave 3 votes
HMSL-06, GAP-P4, DETOX-P01
S–wave 3 votes
HMSL-06, S40RTS, savani
P–wave 3 votes
HMSL-06, MITP_2011, DETOX-P01
S–wave 3 votes
S40RTS, SEMUCB-WM1 and savani
P–wave 3 votes
GAP-P4, MITP_2011, DETOX -P01
S–wave 3 votes
HMSL-06, SEMUCB-WM1, savani
Percentage of surface area (%)

Supplementary Figure 6 (previous page)

Comparison of the vote map procedure for alternative combinations using 3 of the 4 alternative tomography models used in the manuscript, for Vp (solid) and Vs (dashed). The combination models are listed within each panel. The surface area is the coverage for the (maximum) 3 votes, and is complementary to Figure 1b and 1c which shows the maximum 4 votes. Fast velocities are shown in the top panels (a) and slow velocities in the lower panels (b). While there is some variability between the combinations, the observed decorrelation between P and S-wave velocity models is consistent at ~1400 km for fast velocities and ~1800 km for the slow velocities. This suggests that, regardless of which three models are used (conversely, the one model which is excluded), the effects of the iron spin cross-over in ferropericlase can be observed.

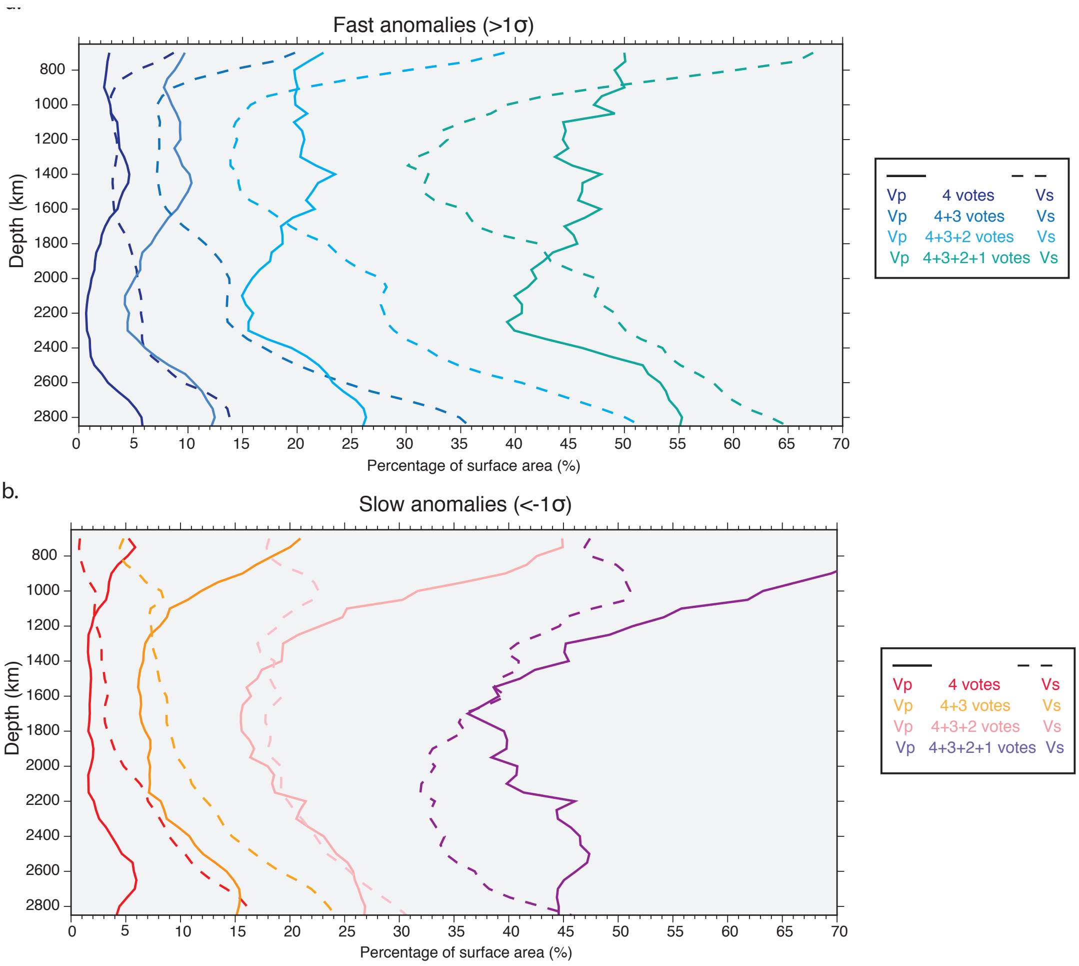

Supplementary Figure 7

Comparison of depth-dependent changes in surface area for alternative vote combinations, Vp (solid) and Vs (dashed). In Figure 1, only the area corresponding to the maximum vote of 4 was shown; "4+3 votes" indicates that the area corresponding to votes of 3 and 4 are summed and plotted, "4+3+2" indicates votes of 2, 3 and votes are summed and plotted etc. Fast velocities are shown in the top panels (a) and slow velocities in the lower panels (b). The vote counts are listed in the inset panel. For the combination of 4 and 4+3 votes (and 4+3+2 for the fast anomalies), the decorrelation signal is very similar. However, when 2 and 1 votes are also added the signal becomes more complicated; this is due to the potential inclusion of noise/artefacts which may not be robust features (because only one model captured it; see Shephard et al. (2017)[4] for further details). Nonetheless, the robustness of the signal suggests that the effects of the iron spin cross-over can be observed.

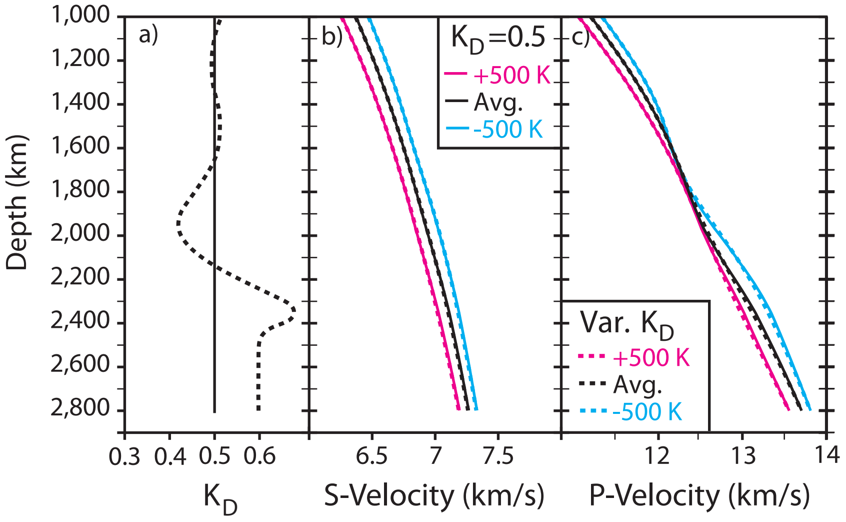

Supplementary Figure 8

The change in the seismic velocities using constant versus depth dependent partitioning of iron between Br and Fp, K_D. Panel (a): Depth-dependent K_D [5] curve (dashed line) and the constant 0.5 value (solid line) used in the main text. Panels (b and c): Shear and compressional velocities for the depth-dependent K_D [5] case (dashed lines) and the constant value 0.5 case (solid lines, same as Figure 2). A higher proportion of Fe in the ferropericlase (lower K_D) may increase the crossover pressure[6]. We find that the FeO content in ferropericlase remains below 25 mol%, which is the threshold for observing substantial increases in the crossover transition pressure[7] for the depth-dependent K_D case. Thus, depth-dependent K_D does not have a significant influence on the crossover depth/pressure range over which we observe the anomalous signal in compressional velocity in the tomographic models.

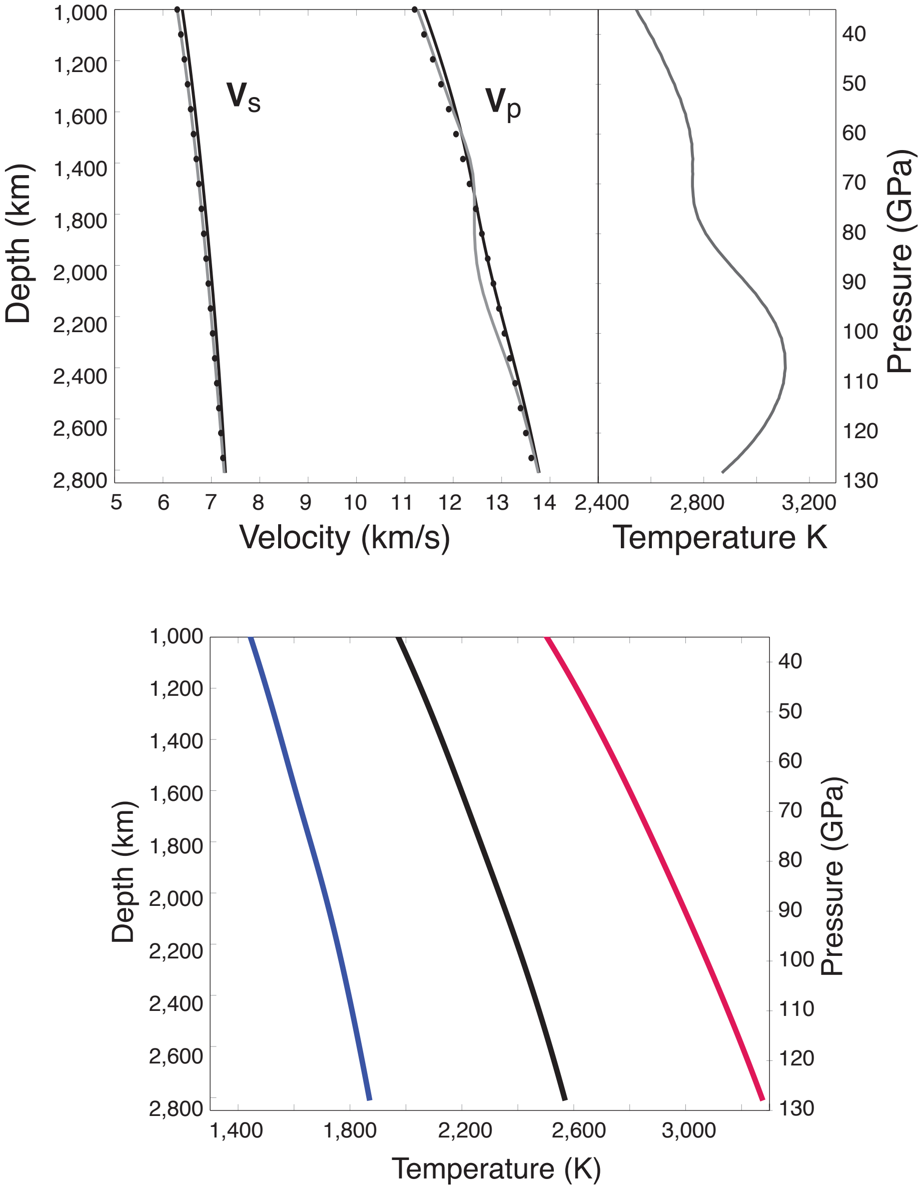

Supplementary Figure 9

Plots related to velocity, temperature and pressure/depth calculations. Top panel: Development of Figure 1d. PREM[8] is shown in black circles and the black lines are the calculated velocities for pyrolite[9]. Figure 1d demonstrates the spin transition effect on Vp for the case in which predicted Vs matches PREM (grey lines). Since Vs for pyrolite does not fit PREM with an adiabatic temperature gradient[10, 11], the temperature profile that shifts Vs to align with PREM (grey line right panel) undulates in the lowermost mantle. Bottom panel: The self-consistent geotherms from our pyrolite calculations[9] for the elastic moduli and velocity profiles plotted in Figure 2. The calculations start by setting the temperature at the top of the lower mantle to 1373 K (blue, the -500 K case), 1873 K (black, the average case), and 2373 K (red, the +500 K case) and allowing the temperature to increase adiabatically as the calculations proceed to higher pressures across the lower mantle.

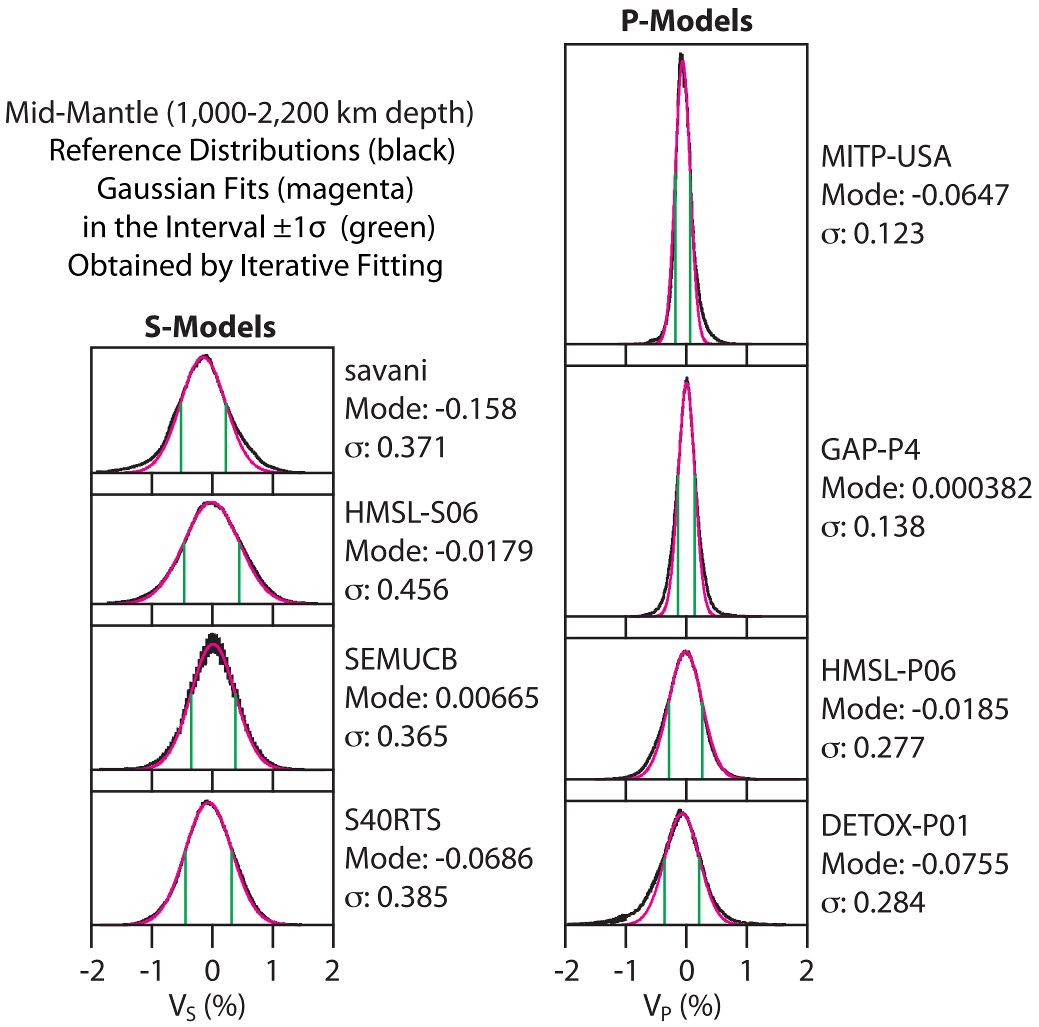

Supplementary Figure 10

The results of our Gaussian-fitting procedure (see Methods) for all 8 tomography models used in this study. Analysis of velocity-frequency distributions of a variety of tomographic models reveals that they exhibit significant differences that confound intermodal comparisons[12]. These differences can be categorized as scale/amplitude (e.g., caused by variability in tomographic model data, design, regularization), shift/alignment (e.g., caused by reference to different 1-D global models), and shape of the distributions (variations in distribution morphology that remain even after accounting for linear shift and scale differences). By analyzing distributions we find that all models yield Gaussian-like variations in Vp and Vs in the depth range 1,000-2,000 km, however, there are particularly large discrepancies in amplitude between the different models[12]. These scale differences must be normalized to a reference standard in order to establish a useful definition for fast and slow anomalies that can be compared across the suite of models. We do this by combining each model from 1,000-2,200 km depth, and performing iterative Gaussian fitting to the central portion (i.e., within ±σ) of the resultant distribution as described in Methods. The value of σ obtained in this manner is then used to define what qualifies as fast and slow anomalies in the models).

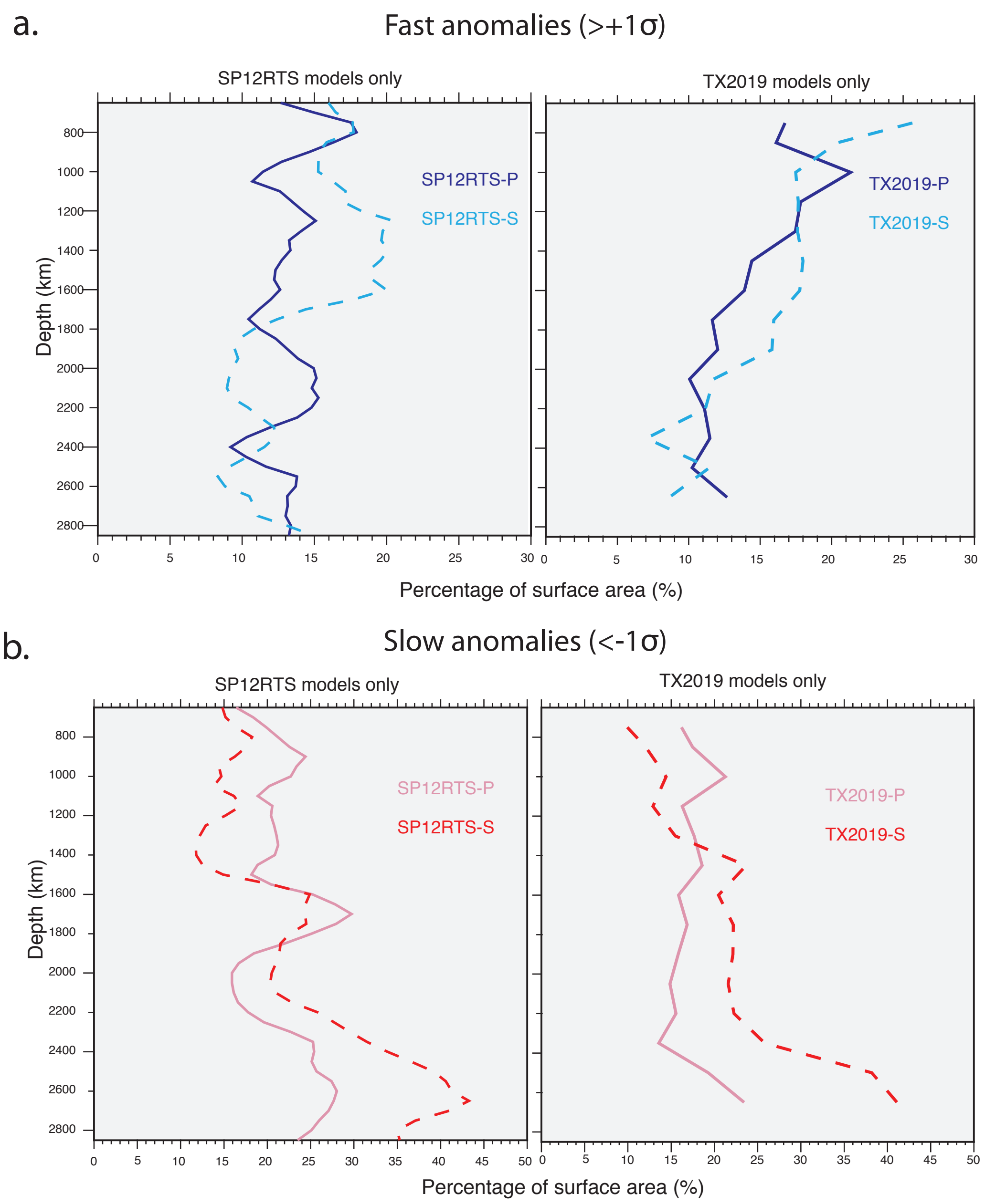

Supplementary Figure 11

Surface area calculations for two additional tomography models. In addition to the eight models used in the paper, depth-dependent change in surface areas for the joint tomography models of SP12RTS[13] and TX2019[14] are also included for reference. The trend between P- and S-wave models is somewhat variable between the individual model pairs of SP12RTS, TX2019, (as for HMSL[3], Supplementary Figure 5) but do hint at a mid-mantle decorrelation similar to the models analyzed in the main text. However, aspects of their construction such as inclusion of subducting slabs in the starting model for TX2019 and the long-wavelength SP12RTS make them less ideal for the mid-mantle focus in this study.

Supplementary References

1. Shukla, G. et al. Thermoelasticity of Fe^{2+}-bearing bridgmanite. Geophys. Res. Lett. 42, 1741– 1749 (2015).
2. Wu, Z., Justo, J. & Wentzcovitch, R. Elastic anomalies in a spin-crossover system: Ferropericlase at lower mantle conditions. Phys. Rev. Lett. 110, 228501 (2013).
3. Houser, C., Masters, G., Shearer, P. & Laske, G. Shear and compressional velocity models of the mantle from cluster analysis of long-period waveforms. Geophys. J. Int. 174, 195–212 (2008).
4. Shephard, G., Matthews, K., Hosseini, K. & Domeier, M. On the consistency of seismically imaged lower mantle slabs. Science Reports 7: 10976 (2017).
5. Piet, H. et al. Spin and valence dependence of iron partitioning in Earth's deep mantle. Proc. Nat. Acad. Sci. 113 (40), 11,127–11,130 (2016).
6. Lin, J.-F., Speziale, S., Mao, Z. & Marquardt, H. Effects of the electronic spin transitions of iron in lower mantle minerals: Implications for deep mantle geophysics and geochemistry. Reviews of Geophysics 51, 244–275 (2013).
7. Persson, K., Bengtson, A., Ceder, G. & Morgan, D. Ab initio study of the composition dependence of the pressure-induced spin transition in the $(Mg_{1-x},Fe_x)O$ system. Geophys. Res. Lett. 33, L16306 (2006).
8. Dziewonski, A. & Anderson, D. Preliminary reference Earth model. Phys. Earth Planet. Inter. 25, 297–356 (1981).
9. Valencia-Cardona, J. et al. Influence of the iron spin crossover in ferropericlase on the lower mantle geotherm. Geophys. Res. Lett. 44, 4863–4871 (2017).
10. Houser, C., Hernlund, J., Valencia-Cardona, J. & Wentzcovitch, R. Discriminating lower mantle composition. Phys. Earth Planet. Int. 308, 106552 (2020).
11. Cobden, L. et al. Thermochemical interpretation of 1-D seismic data for the lower mantle: The significance of nonadiabatic thermal gradients and compositional heterogeneity. J. Geophys. Res. 114, doi:10.1029/2008JB006262 (2009).
12. Hernlund, J. & Houser, C. On the distribution of seismic velocities in Earth's deep mantle. Earth Planet. Sci. Lett. 265, 423–437 (2008).
13. Koelemeijer, P., Ritsema, J., Deuss, A. & van Heijst, H.-J. SP12RTS:A degree-12 model of shear- and compressional-wave velocity for Earth's mantle. Geophysical Journal International 204, 1024–1039 (2016).
14. Lu, C., Grand, S. P., Lai, H. & Garnero, E. J. TX2019slab: A new P and S tomography model incorporating subducting slabs. Journal of Geophysical Research: Solid Earth 124, 11549–11567 (2019).
